# Rare Variants in *MYD88*, *IRAK4* and *IKBKG* and Susceptibility to Invasive Pneumococcal Disease: A Population-Based Case-Control Study

**DOI:** 10.1371/journal.pone.0123532

**Published:** 2015-04-17

**Authors:** Magda K. Ellis, Katherine S. Elliott, Anna Rautanen, Derrick W. Crook, Adrian V. S. Hill, Stephen J. Chapman

**Affiliations:** 1 Wellcome Trust Centre for Human Genetics, University of Oxford, United Kingdom; 2 Queensland Institute of Medical Research, Brisbane, Australia; 3 Centenary Institute and Sydney Medical School, University of Sydney, Sydney Australia; 4 Department of Microbiology, John Radcliffe Hospital, Oxford, United Kingdom; 5 Oxford Centre for Respiratory Medicine, Churchill Hospital Site, Oxford University Hospitals, Oxford, United Kingdom; University of Cambridge, UNITED KINGDOM

## Abstract

Although rare variants within the Toll-like receptor signalling pathway genes have been found to underlie human primary immunodeficiencies associated with selective predisposition to invasive pneumococcal disease (IPD), the contribution of variants in these genes to IPD susceptibility at the population level remains unknown. Complete re-sequencing of *IRAK4*, *MYD88* and *IKBKG* genes was undertaken in 164 IPD cases from the UK and 164 geographically-matched population-based controls. 233 single-nucleotide variants (SNVs) were identified, of which ten were in coding regions. Four rare coding variants were predicted to be deleterious, two variants in *MYD88* and two in *IRAK4*. The predicted deleterious variants in *MYD88* were observed as two heterozygote cases but not seen in controls. Frequencies of predicted deleterious IRAK4 SNVs were the same in cases and controls. Our findings suggest that rare, functional variants in *MYD88*, *IRAK4* or *IKBKG* do not significantly contribute to IPD susceptibility in adults at the population level.

## Introduction

*Streptococcus pneumoniae* is the leading cause of community-acquired pneumonia worldwide and remains a major cause of death in children under five years of age [1]. The predominant cause of death resulting from *Streptococcus pneumoniae* infection arises from invasive pneumococcal disease (IPD), defined as the isolation of bacteria from a normally sterile site. Nasopharyngeal carriage of the bacteria is widespread in the population, with most individuals harbouring the pathogen at some point without adverse consequence and only a small minority of individuals succumbing to invasive disease. It is now widely accepted that host genetics plays an important role in determining an individual’s risk of infection with a number of pathogens, including *Streptococcus pneumonia* [2,3]. A number of studies have attempted to identify the specific genetic loci that influence susceptibility to IPD, largely through investigation of common polymorphisms in biologically plausible candidate genes [4,5,6,7,8,9,10]. In keeping with other complex diseases, the size of effect of these polymorphisms is relatively small and it is highly likely that additional genetic variation makes a substantial contribution to IPD susceptibility. Multiple causative rare genetic variants of individually large effect size are widely considered a possible source of the ‘missing heritability’ of common human disease [11], and candidate gene resequencing studies have successfully identified a number of rare variants underlying complex disease phenotypes [12]. This increasing interest in rare variants in susceptibility to complex disease is particularly relevant to conditions that exert strong evolutionary selective pressure, such as bacterial disease. The huge burden of childhood disease resulting from pneumococcal infection suggests that most major IPD susceptibility variants are likely to be of recent origin and individually rare.

Further support for a major role of rare variants in susceptibility to IPD comes from recent insights into the nature of primary immunodeficiency (PID). Until recently, PIDs were considered solely to consist of mutations in multicase families leading to recurrent and diverse childhood infections, whereas now the term also encompasses newly described mutations with incomplete penetrance leading to sporadic, common and sometimes selective infectious diseases in otherwise healthy adults [13]. Of particular relevance to bacterial disease has been the identification of novel PIDs involving the Toll-like receptor (TLR)-Nuclear Factor-B (NF-B) signalling pathway, which plays a critical role in the early recognition of invading microorganisms and the initiation of an inflammatory host response. Causative mutations within four genes in the TLR-NF-κB pathway have been described in association with IPD: *IKBKG* (inhibitor of kappa light polypeptide gene enhancer in B-cells, kinase gamma, which encodes the protein NEMO), *NFKBIA* (nuclear factor of kappa light polypeptide gene enhancer in B-cells inhibitor, alpha), *IRAK4* (interleukin-1 receptor-associated kinase 4) and *MYD88* (myeloid differentiation primary response gene 88) [14,15,16]. The impact of these variants on immune signalling pathways differs: *IKBKG* and *NFKBIA* mutations interrupt multiple innate and adaptive pathways which signal to NF-κB, including the TLR pathway, whereas mutations in *IRAK4* and *MYD88* appear to disrupt only TLR and interleukin-1 receptor signalling [17]. Consistent with this, the immunodeficiency resulting from *IKBKG* and *NFKBIA* mutations is typically severe and broad spectrum, encompassing susceptibility to a wide range of pathogens including encapsulated pyogenic bacteria, mycobacteria, fungi and viruses, and frequently associated with the developmental disorder anhidrotic ectodermal dysplasia (AED) [14,18]. IRAK4 and MYD88 deficiencies on the other hand appear to result in a narrow-spectrum immunodeficiency characterised by IPD and the absence of AED [13,19,20].

The observation that frequently the sole manifestation of IRAK4 or MYD88 deficiency is an increased susceptibility to IPD raises the possibility that multiple, diverse, individually rare mutations in *IRAK4* or *MYD88* may significantly impact on susceptibility to this disease in the general population. Furthermore, subgroups of individuals with NEMO deficiency resulting from *IKBKG* mutations do not demonstrate evidence of AED and may exhibit susceptibility to pyogenic bacterial infection without apparent vulnerability to mycobacteria or viruses [21]. Only a very small number of individuals with these PIDs have been described to date world-wide, however, and their overall contribution to IPD susceptibility is unknown. In order to define the possible contribution of rare variants in these genes to IPD susceptibility at the population level, we performed complete re-sequencing of *IRAK4*, *MYD88* and *IKBKG* in IPD cases and population-based controls.

## Materials and Methods

### Study patients

The IPD sample collection has been previously described in detail [9]. Briefly, blood samples were collected from patients with IPD (defined by the isolation of *Streptococcus pneumoniae* from a normally sterile site) as part of an enhanced active surveillance program in three hospitals in Oxfordshire, United Kingdom (John Radcliffe, Horton General, and Wycombe General Hospitals). DNA samples were assessed for quality by gel electrophoresis and PCR ability using two optimised long range PCRs of 4kb in length. Of the available IPD samples, 164 samples were of sufficient DNA quality to permit sequencing. Although all patients had invasive disease, their initial clinical presentations were as follows: pneumonia, 62.4%; isolated bacteremia, 11.2%; meningitis, 10.40%; pneumococcal empyema 8.9% and other presentations, 7.2%. Nine patients (5.8%) were children under the age of 18 years of age and 53% of cases were male.

### Control samples

The control group comprised a subset of the United Kingdom Blood Service (UKBS) biobank samples previously described in the WTCCC GWAS studies [22]. All DNA samples in the control group were extracted from whole blood. Only samples originating from individuals of European origin living in the South of England (the same location within the UK as the case subjects) were selected for this study. Following tests for genomic quality and PCR ability, 164 control DNAs were selected for pooling. The controls ranged in ages from 15–65 (mean and median ages of 42.9 and 45, respectively) and comprised 50% males.

### Pooling strategy

All case and control DNA samples were quantified using the Picogreen dsDNA quantification kit (Invitrogen) and normalised to 10ng/ul. Samples were combined into four pools of cases and four pools of controls each comprising equal amounts of DNA from 41 individuals.

### Target amplification and PCR

Primers were designed to amplify the complete gene and promoter region for *IRAK4*, *MYD88*, and *IKBKG*. A total of 16 amplicons were used to span each gene; flanking amplicons overlapped by 40-60bp. Amplicons were verified using the DNA 7500 and 12000 chips on a bioanalyser and quantified by picogreen. Final amplicons were diluted to the same molar concentration and pooled. Library preparation and subsequent re-sequencing was performed by the Wellcome Trust Centre for Human Genetics, Oxford, Sequencing core.

### Sequencing strategy and bioinformatics

The case samples were sequenced using the Illumina GAII platform in single-end reads. Control samples were bar-coded and sequenced in multiplex on the Illumina HiSeq using paired-end reads. Allele ratios contributing to the sample pools were estimated using syzygy software (http://www.broadinstitute.org/software/syzygy [23]). The output file containing all variants is provided as supporting information in S1 Dataset. Sequencing reads were visualised using Integrative Genomics Viewer (IGV) software [24,25].

### Statistical analyses

Association tests between allele frequencies and accumulative variants [26] were analysed using Fisher’s exact and Chi square tests where appropriate with nominal statistical significance determined at 0.01. Data was compared to the 1000genomes phase 1 data [27] using only available data from Great-Britain (1000g-GBR). In addition, if variants appeared to be novel, dbSNP [28] and Exome Variant Server (EVS)[29] were also checked for further confirmation. The functional consequence of missense, splice-site and nonsense variants were determined using Polyphen2, [30] SIFT [31] and mutationTaster [32] softwares.

### Ethics statement

The study has been approved by the Central University Research Ethics Committee, University of Oxford (MSD-IDREC-C1-2014-112) which approved not obtaining consent. Consent was not required, and thus not obtained, as these samples were anonymised and collected prior to the UK Human Tissue Act 2006 in the initial collection of the samples. Nor was consent obtained from next of kin, caretakers, or guardians on behalf of the minors/children enrolled in this study. All samples were anonymised and de-identified prior to analysis.

## Results

In the final dataset, we identified a total of 233 single-nucleotide variants (SNVs) within these three genes, of which 170 SNVs were observed in cases and 166 were observed in control sample data.

Most variants were found to be intronic (86.7%) or in UTR-regions (9.0%) as shown in Table 1. Testing for associations was performed for all individual SNVs in these non-coding regions. After manual filtering for repetitive sequences, and the confirmation of the mutation in dbSNP and/or 1000genomes if the variant was only observed in the control group, only one common variant (rs4251513) in *IRAK4* was found to be associated with IPD at the 0.05 significance level (p = 9.96x10^-3^; OR = 1.498; 95% CI: 1.101–2.037). This polymorphism did not retain statistical significance following correction for multiple testing. This variant was found to be intronic with an allele frequency of 0.543 and 0.442 in cases and controls, respectively.

**Table 1:** Single nucleotide variant types identified in *MYD88*, *IRAK4* and *IKBKG*.

	*MYD88*			*IRAK4*			*IKBKG*			TOTAL		
Variant type	Cases	Controls	Total	Cases	Controls	Total	Cases	Controls	Total	Cases	Controls	Total
MS/NS/SS^a^	2	1	3	3	4	4	0	1	1	5	6	8
Synonymous	1	0	1	1	1	1	0	0	0	2	1	2
Intron	5	3	6	130	107	157	15	33	39	150	143	202
3'UTR	2	3	3	6	11	12	0	0	0	8	14	15
5'UTR	0	0	0	1	1	2	4	1	4	5	2	6

^a^MS/NS/SS: Missense/Nonsense/Splice-site

Nine SNVs (3.4%) were identified in coding regions; these comprised two synonymous, six missense and one nonsense variants. In addition, one donor splice site variant was also observed. All missense, nonsense and splice site variants identified are shown in Table 2. Only one variant (rs4251545) was found to have an allele frequency of greater than 5% in both the case and control groups. This variant was not predicted to have an effect on protein function and was not statistically different between the two groups (p = 0.354). All remaining mutations were rare, with allele frequencies of less than 1% in controls. Four rare coding variants were predicted to be deleterious: two variants in *MYD88* and two variants in *IRAK4*.

**Table 2:** Non-synonymous (missense), splice site (SS) and nonsense variants identified in IPD cases and controls.

									IPD	UKBS	1000g-GBR	ALL-UK	
chr	Position	rs id	ref	alt	gene	function	Coding	Prediction	N^a^(MAF)^b^	N(MAF)	N(MAF)	P-value	OR (95% CI)
3	38182062	rs148149492	T	C	*MYD88*	missense	L229S	damaging	1 (0.003)	0 (0.000)	0 (0.000)	0.393	0 (NA)
3	38182087	*de novo*	C	T	*MYD88*	donor SS	-	damaging	1 (0.003)	0 (0.000)	0 (0.000)	0.393	0 (NA)
3	38181392	E_38181392^a^	G	T	*MYD88*	missense	Q135H	benign	0 (0.000)	1 (0.003)	0 (0.000)	1	0 (NA)
12	44172041	rs121908002	C	T	*IRAK4*	nonsense	Q293X	damaging	1 (0.003)	1 (0.003)	0 (0.000)	1	0.650 (0.040–10.390)
12	44177511	rs55944915	G	A	*IRAK4*	missense	R267H	damaging	3 (0.009)	3 (0.009)	3 (0.017)	0.685	0.650 (0.130–3.221)
12	44180295	rs4251545	G	A	*IRAK4*	missense	A304T	benign	35 (0.107)	28 (0.086)	14 (0.079)	0.271	1.325 (0.827–2.124)
12	44180504	*de novo*	A	T	*IRAK4*	missense	M333L	benign	0 (0.000)	1 (0.003)	0 (0.000)	1	0 (NA)
X	1.54E+08	rs189237568	C	T	*IKBKG*	missense	R50C	benign	0 (0.000)	2 (0.006)	0 (0.000)	0.523	0 (NA)

^a^ Number of alleles counted for each variant

^b^ Minor allele frequency

^c^ Identified in the EVSdatabase in EuroAmerican populations with a MAF of 0.000233

Of the *MYD88* variants, the rs148149492 mutation causes an amino acid change at L229S. Intriguingly, this amino acid substitution is not itself predicted to be damaging, however in a number of *MYD88* transcript splice variants, this mutation is predicted to interfere with the splicing and acts as a donor splice variant. The second *MYD88* variant observed in the data was novel and predicted to interfere with mRNA splicing of an alternate splice form of *MYD88* within the same intronic region as rs148149492. In the major isoform, both mutations lie within the TIR-domain of *MYD88* and thus have the potential to disrupt MYD88-TIRAP activity [33]. One mutant allele for each of these two damaging splice site variants were found in the IPD cases, in different sequencing pools, but they were not observed in the controls (Table 2). A further *MYD88* coding variant was identified in the control group that was not observed in the case group. This Q135H substitution was only observed once and predicted to have a benign effect on protein function. Neither variant was individually associated with IPD when comparing the cases to all UK controls combined (Table 2). Testing for mutational load by summing the putatively pathogenic alleles in all groups also failed to show an association with IPD compared to all UK data combined (p = 0.154).

The two variants observed in *IRAK4* which were predicted to be deleterious were observed in both IPD cases and the UKBS control group with identical frequencies (Table 2). The first mutation, G293X, has been previously described in children with IRAK4 deficiency with a predisposition to recurrent IPD [13] when observed in homozygous states or as a compound heterozygote with another rare and deleterious IRAK4 allele. In our study only a single heterozygote form was observed in both the IPD cases and controls. Furthermore no other rare alleles were present in the pool in which this heterozygote was observed, thereby excluding the possibility of a compound heterozygote in this case. While the *IRAK4* R267H variant was observed at a slightly higher frequency (0.9%), this mutation was also observed in equal frequencies between the case and controls groups and is also present in the 1000genomes data with a frequency of 1.7%. No association for this allele was observed between cases and all UK control data combined (p = 0.685; OR = 0.650; 95% CI: 0.130–3.221) as shown in Table 2. Mutational load of putatively damaging variants was also not found to be significant in IRAK4 (p = 1.000; OR = 1.363; 95% CI: 0.330–3.913).

## Discussion

The identification of rare pathogenic variants in TLR pathway genes underlying episodes of invasive pneumococcal disease has significantly advanced understanding of human host defence against the pneumococcus and signalled a paradigm shift in the concept of primary immunodeficiency [13,16,19,34]. A key question following these advances relates to the genetic architecture of IPD susceptibility in the general population and the specific contribution of rare mutations in *IRAK4*, *MYD88* and *IKBKG* to this phenotype. Here we demonstrate that rare, deleterious variants in these genes are unlikely to make a major contribution to IPD in the general population.

None of the *IKBKG* mutations known to cause NEMO deficiency [14] or indeed any other putatively deleterious novel *IKBKG* mutations were observed in IPD cases in our study. While two alleles of the R50C coding variant were observed in this study (Table 1), this variant was predicted to be benign and was only observed in the control group, suggesting that it has little influence on NEMO expression or activity.

The only mutation identified in this study that has previously been implicated in susceptibility to IPD was *IRAK4* Q293X. However, only patients homozygous or compound heterozygous for this mutation have been reported in previous studies of primary immunodeficiency [13]. In addition, the observation of a heterozygote in the UKBS control group suggests that heterozygous carriage of this mutation has little impact on susceptibility. The putatively deleterious *IRAK4* mutation R267H was also found to have a similar frequency in cases and the UKBS and in the 1000genomes, again suggesting that this variant has no major impact on the risk of IPD.

Two predicted deleterious variants were identified in this study in *MYD88*. Both of these variants were observed in cases but not in the control group, and their population frequency was also found to be extremely low in the 1000genomes database (0.0009 and 0 for rs148149492 and the novel variant, respectively). The primary immunodeficiency associated with MYD88 deficiency is known to be autosomal recessive however [19], and the two mutations in this study were observed in different case pools and therefore represent two heterozygous individuals, rather than a single, compound heterozygote individual with both mutations. We are unable to exclude the possibility that heterozygous carriage of these deleterious variants may result in a partial MYD88 deficiency and have contributed to IPD susceptibility in these two individuals, but a significantly larger study would be required to demonstrate this. MYD88 has two important protein features comprising the death domain (DD) and its TIR (Toll IL-1R) domain which are required for IRAK4 and IL-1R interaction respectively [15]. Given both mutations identified in this study were located in the TIR domain they may reduce the ability of MYD88 to interact with IL-1R. Furthermore, both mutations may impact on regulation of alternate splicing of MYD88, which has been shown to influence TLR and IL-1 receptor signalling [17]. The effect of these SNVs on protein structure and activity and how they may contribute to susceptibility requires further investigation.

A limitation of this study relates to sample size, which reflects the challenge in assembling DNA collections from well-phenotyped patients with a rare, acute and severe disease. Our sample size is powered to identify associations with odds ratios of 12 and greater with a population (control) allele frequency of 0.005, at p = 0.01 significance level with 80% power (calculated using the Genetic Power Calculator [35]). This calculation reflects testing of individual variants however and may underestimate power compared to our testing of the mutational load of predicted functional variants. Although significantly larger sample sizes are recommended for exome sequencing studies aimed at identifying rare variants in association with complex disease [12], this is unlikely to be applicable to our candidate gene sequencing study of IPD which represents an extreme phenotype with a very low population incidence [36]. Although the effect size of genetic risk variants for severe bacterial disease is currently unknown, the only reported invasive bacterial disease case-control study of candidate gene sequencing used a comparable sample size (230 cases) and reported an odds ratio of 27.0 (*P* = 2x10^-6^) for the effect of combined *TLR4* coding variants on susceptibility to invasive meningococcal disease [37]. Furthermore, the low background incidence of IPD [36] suggests that the subsequent development of disease in controls would be exceptionally rare and hence such ‘misclassification’ of individuals as controls is unlikely to impact further on our study power.

Previous studies describing the role of rare variants in IPD have primarily investigated recurrent disease in children and identified a number of gene variants in association with this phenotype. Indeed, a recent study investigating immunological deficiencies in 163 children with IPD reported that 26% of cases had an underlying primary immunodeficiency (PID) although only one child had an apparent TLR-NF-kB pathway defect (MyD88 deficiency) [38]. A study which examined TLR signalling from an immunological perspective failed to show evidence of TLD defects in a smaller sample size of 50 children with IPD. The limited evidence for a major role of rare variants in *MYD88*, *IRAK4* or *IKBKG* in IPD in the current study may reflect in part the higher median age group (58 years of age) in our cohort and the single-episode phenotype (as opposed to recurrent disease). However, alternative monogenic mechanisms within the TLR-NFkB pathway may exist and warrant further investigation through both genetic and immunological assays in order to explore the potential role of PIDs underlying adult IPD. The observation that the immunodeficiency of IRAK4- and MYD88-deficient children usually improves with age [29] suggests a gradual compensation of the adaptive immune response with time or that the innate immune response is able to mature with age. Furthermore, non-TLRs may govern alternative mechanisms of the immune response in pathogen recognition and should also be considered in future genetic studies of adult IPD.

## Conclusions

In conclusion, our findings suggest that rare, functional variants in *MYD88*, *IRAK4* or *IKBKG* are of little relevance to the phenotype of IPD typically seen in clinical practice. Although the identification of primary immunodeficiencies associated with these genes has led to major insights into human immune defence against the pneumococcus, they may only be of direct clinical relevance to a small minority of individuals. The relative contributions of common polymorphism and rare genetic variants to IPD susceptibility at both the individual and population level remain unknown, although the widespread application of next generation sequencing has the potential to define the genetic architecture of susceptibility to bacterial disease in humans.

## Supporting Information

S1 Dataset Dataset. (XLS)
